# Supplementary material for: Beyond shallow feelings of complex affect: Non-motor correlates of subjective emotional experience in Parkinson’s disease
Source: PLoS One. 2023 Feb 24;18(2):e0281959. doi: 10.1371/journal.pone.0281959 (PMC9955984; doi:10.1371/journal.pone.0281959)
Supplement: Attachment — Submitted filename: Response to Reviewer.docx [file pone.0281959.s001.docx]

**Response to Reviewer’s comments**

**General comments**

In an important inquiry, the authors explore the lesser recognised non-motor symptoms of Parkinson’s disease. While there is a coherent aim, and data collected support this aim, there are some flaws in the attempts to tease apart the more nuanced subjective emotional dimensions which weaken the conclusions drawn. There is an interesting but not particularly strong discussion.

**Answer:**

The authors would like to take this opportunity to sincerely thank the Reviewer for the thorough review of the manuscript and for the critical and very helpful comments, which contributed to improve the overall quality of the present manuscript. We have now revised the statistical analysis to ensure that appropriate conclusions can be drawn. The Discussion was also revised and updated following the specific recommendations given by the Reviewer.

**Comment 1:** In pursuit of understanding how Parkinson’s affects emotion processing, with an interest in autonomic dysfunction and a potential relationship between the two, there are several striking findings:

1. Lower concordance between target emotion and reported emotion in PD in response to video clips intended to elicit tenderness.
2. Reduced emotional intensity for amusement and tenderness in PD, but not the others, suggesting—and well explored in the discussion—that there is a complexity element to consider.
3. More ‘neutral state’ responses by people with PD than in healthy controls (though no means are reported)
4. No significant LEDD correlation with emotion responses suggesting emotion processing (for film clips at least!) could be a dopamine independent process

**Answer:**

*Descriptive statistics for the neutral state responses were now added.*

Lines 164-166 (Results)

On the whole, patients reported a higher rate of no emotional experience (Neutral State) to an affective context (22% in the PD group *vs.* 11% in the control group; χ^2^ = 8.30, *p =* 0.004).

**Comment 2:** While these findings are interesting, worthy of attention and important, there is a significant flaw in the analysis that prevents me drawing any useful conclusions. In particular, sex is a founding factor that hasn’t been properly addressed. While a subgroup is established to address the factors like age and sex, one group has 28% female and the other 65%. With the literature the authors cite about the differences in female/male subjective emotion measures, this could well explain the group differences more so than Parkinson’s disease itself. Unfortunately, I can see no evidence of the authors including sex in part of their analysis as a covariate or in a secondary comparison to rule out this confound, and I am far from convinced about the use of the PD subgroup to address these factors.

**Answer:**

We acknowledge the potential effect of sex on the study findings. After carefully revising this concern, taken together, the following aspects support that there is not a significant effect of sex on our results:

1. Although the PD patients were predominantly males, **the differences in sex distribution between the PD and control groups were not statistical significant**, *p* = 0.06. (Results, lines 139 - 140)
2. The incapacity to experience Tenderness in the PD group occurred regardless of participant’s sex or sex distribution (predominantly males): **80% of females and 69% of males exhibited an impaired experience of Tenderness** (χ^2^ = 0.02, *p =* 0.90). (Results, lines 159 -161)
3. In addition, we included the “sex” of participants as an independent variable in regression models to explore whether the sex differences explained the variations of the self-reported emotion ratings, and **no significant effect of “sex” on any feature of the subjective emotional experience was found**, *e.g.*, sex effect on ratings of Tenderness intensity: t = 0.53, ***p = 0.60***; sex effect on ratings of Amusement intensity: t = -1.14, ***p* = 0.26**). (Results, lines 170 -172)

**Comment 3:** There are a few other points I provide feedback on, but this (*comment 2*) is a significant flaw I believe needs addressing before going further. Overall, it is an interesting and important investigation, and difficult data to effectively analyse given the diversity of the PD population and their on-medication state (which I do support as a much kinder scenario in which to work with people with PD). I am not convinced this is an effective approach to understand the emotional and autonomic alterations in Parkinson’s disease.

**Answer:**

In this study, we aimed at assessing the subjective emotional experience in PD. We also explored the association of self-reported emotional ratings with the self-reported SCOPA-AUT scores, to consider features which are similar in nature as they account for the *subjective component* of PD complaints across the emotional and autonomic dimensions.

**Comment 4:** Before providing points on specific lines below, I share briefly some further ideas and feedback I hope are of some use.

Firstly, I would be interested to see data on what emotions were selected by participants when concordance rates were low. For example, was tenderness concordance low in PD because of more neutral state responses, or because of high selection of non-target emotions?

**Answer:**

Lines 157 – 164 (Results)

PD patients experienced Tenderness significantly less often than controls (53%, Figure 1), and frequently showed no emotional reactivity (Neutral State, 19%) to stimuli displaying tender cues. (…) Several patients felt Sadness (11%), Amusement (11%) or Anger (6%) in response to tender scenes, which differed significantly from the overall rate of emotional responses other than Tenderness experienced by the healthy participants in the same affective setting, excluding Neutral State (16% in the PD group *vs.* 7% in the control group; χ^2^ = 4.16, ***p =* 0.04**).

Lines 281 – 290 (Discussion)

However, our findings support the possibility that patients unable to experience Tenderness, may generate alternative affective responses that demand less cognitive efforts or recruit preserved affective territories. Sadness and Anger, reported by 17% of patients in response to film excerpts intended to elicit Tenderness, are both dimorphous emotions (*i.e.*, the normative and contextual interpretation are opposing in valence) that communicate positive affective states when displayed in positive contexts that represent emotionally evocative situations [38]. This de-complexification of the emotional response may result from the integration of the limited cognitive resources available to process affect, as a coping strategy that compensates for the deficits associated with the experience of Tenderness, which in turn leads to a more restricted emotion inventory.

**Comment 5:** The dissociation of tenderness and amusement across concordance and intensity is interesting.

**Answer:**

Lines 275-281 (Discussion)

Tenderness and Amusement are both complex positive emotions, though they differ in affective motivation, a basic dimension of human emotion [34]. Tenderness can be regarded as a high-approach motivational emotion, whereas Amusement is a low-approach motivational emotion [35]. Decreased approach motivation and diminished emotional response are both symptoms of apathy, a behavioral syndrome highly prevalent in PD that might also result from the severe dopamine depletion state in the mesocorticolimbic system, more specifically in the *nucleus accumbens* [36,37].

**Comment 6:** Would also be good (important) to see emotional valence data reported, even if just group means for positive and negative emotions.

**Answer:**
Lines 174-178 (Results)

Emotional valence ratings were not significantly different between groups (p > 0.07), although pleasant emotions (mean valence ± SD, 6.1 ± 1.2 in PD group vs. 6.7 ± 0.9 in control group; ANOVA F = 3.69, *p* = 0.06) exhibited a greater variation than unpleasant emotions (mean valence ± SD, 1.5 ± 1.0 in PD group vs. 1.4 ± 0.7 in control group; ANOVA F = 0.12, *p* = 0.74).

**Comment 7:** Finding no LEDD correlation with emotion intensity is important, but it would be interesting to see this analysis for what are deemed the more complex emotions of tenderness and amusement, which you already provide a strong basis for (lines 240-267).

**Answer:**

Lines 291-303 (Discussion)

Dopamine has been implicated in reward-related processes and therefore associated with positive affect, though the actual role of the mesolimbic dopaminergic system in positive emotions is still not fully elucidated [39]. In keeping with prior reports [10,31,40], we found no significant impact of dopaminergic medication on subjective ratings of emotional experience, suggesting that the complex feelings of Tenderness and Amusement may further depend on systems of neurotransmitters other than dopamine, all of which might be consistently deranged since the early stages of PD. According to the “Three Primary Color Model of Basic Emotions” theory, emotion is a product of the mixture of the three monoamines dopamine, norepinephrine, and serotonin, which combined in various proportions result in the more complex “higher-order” emotions [41,42]. Diffuse pathology of the dopaminergic, noradrenergic and serotonergic systems underlie several non-motor symptoms in the prodromal and clinical stages of PD [43,44], which may have a significant impact on several aspects of mood, emotion and behavior, including de-complexification of the emotional response.

**Comment 8:** Some parts of the discussion are written as if they were bullet points which helps with brevity, but I personally want to see connections made by contextualising the points made. Rather than just making a factual statement, there’s opportunity to share why the point matters, or what is being implied – theoretically or practically.

**Answer:**

*Following the Reviewer’s comment, we updated the full discussion section.*

Lines 304-316 (Discussion)

The experience of positive emotion is associated with several favorable health and psychological outcomes including longevity, larger social networks, and increased prosocial behavior [45]. Moreover, research has indicated that positive affect can serve adaptive functions in moderating relations between stress and health, given that positive emotion has also an important role in regulating emotional responses to negative events and thus contributing to achieving efficient emotion regulation [46]. Since basic emotions are mostly related to negative affect, the impaired experience of cognitively complex emotions in individuals with PD is essentially associated with deficiencies in processing positivity, with important implications for patient subjective and psychological well-being [47]. Besides, Tenderness and Amusement are positive emotions that promote adaptive responses to the potential for reward, by facilitating complex behavior that fosters communication and social interaction [48]. Thus, deficits in affection and humor may have profound social consequences for PD patients, with subsequent impairment in their interpersonal relationships.

**Comment 9:** Finally, I am not familiar enough with the k-means clustering method to provide a critique of it its implementation. I would, though, want to see some validation metrics to assure the reader it has been applied soundly and I can read into its conclusions.

**Answer:**

In an earlier *PLOS ONE* publication [1], Erro and colleagues (2013) reported validation metrics for clustering PD patients into four distinct phenotypes delineated by the differential involvement of several clinical features. Specific non-motor impairments were prevalent among each of the four patient clusters, namely: cluster 1, severe urinary (autonomic) dysfunction; cluster 2, severe cognitive deficits; cluster 3, intermediate autonomic and mild cognitive impairment; and cluster 4, absence or low autonomic and cognitive involvement. Considering these aspects, we aimed at exploring the non-motor characteristics of *four clusters* of PD patients, profiled according to the affective domain in addition to the *urinary (autonomic) and cognitive dimensions*, which in the present study were found to be associated with the emotional features and in the earlier publication were found to be relevant for the definition of PD subgroups. By applying the cluster analysis as described by Erro and colleagues (non-hierarchical cluster analysis, k-means cluster method, four clusters solution), we obtained consistent results, which extended previous findings to highlight the relevance of the affective symptomatology for the assessment of the non-motor presentations in the PD subtypes.

[1] Erro, R., Vitale, C., Amboni, M., Picillo, M., Moccia, M., Longo, K., Santangelo, G., De Rosa, A., Allocca, R., Giordano, F., Orefice, G., De Michele, G., Santoro, L., Pellecchia, M. T., & Barone, P. (2013). The heterogeneity of early Parkinson's disease: a cluster analysis on newly diagnosed untreated patients. PloS one, 8(8), e70244. <https://doi.org/10.1371/journal.pone.0070244>

**LINE-BY-LINE points**

**Comment 1:** Line 56 – "major role of ANS in the experience of emotions" No reference?

**Answer:** Line 53

*Reference number 13 was added:* Levenson RW (2014) The Autonomic Nervous System and Emotion. Emotion Review 6: 100-112

**Comment 2:** Line 105

Did participants type this response? Did you consider the heavier motor component of this and what effect this might have? Any notable difference in response times?

**Answer:** Lines 101-102

*Yes, they did type the response. Motor effect was not considered. Response time was not a variable considered in the study.*

**Comment 3:** Line 148

complaints not complains

**Answer:** Line 149

*Corrected.*

**Comment 4:** Line 152

Report p value

**Answer:** Line 150

Clinical scores were not significantly associated with age, age at onset, disease duration, or levodopa equivalent daily dose (LEDD) **(*p* > 0.10)**.

**Comment 5:** Line 157-158

This is a key finding I think – apathy has been reported in PD (you have a few refs already along these lines), and higher rates of neutral state responses bolsters the finding of reduced emotional intensity.

**Answer:** Lines 278-281

Decreased approach motivation and diminished emotional response are both symptoms of apathy, a behavioral syndrome highly prevalent in PD that might also result from the severe dopamine depletion state in the mesocorticolimbic system, more specifically in the *nucleus accumbens* [36,37].

**Comment 6:** Line 161

Are these stats reported for tenderness, amusement or both? And if both, why are they grouped together here?

**Answer:**

*Those stats describe the overall PD* ***multivariate effect*** *on the subjective experience of intensity for all emotions (this has been now clarified in the text). Specific statistics for Tenderness and Amusement intensity are reported individually in the Figure 1.*

Lines 168-170

(…) and was significantly reduced for the experience of Tenderness and Amusement (Figure 1; overall PD effect: multivariate ANOVA F = 2.80, *p =* 0.03).

**Comment 7:** Line 163

Report F stat and p value

**Answer:** Lines 174-178

Emotional valence ratings were not significantly different between groups (p > 0.07), although pleasant emotions (mean valence ± SD, 6.1 ± 1.2 in PD group vs. 6.7 ± 0.9 in control group; ANOVA F = 3.69, ***p = 0.06***) exhibited a greater variation than unpleasant emotions (mean valence ± SD, 1.5 ± 1.0 in PD group vs. 1.4 ± 0.7 in control group; ANOVA F = 0.12, ***p = 0.74***).

**Comment 8:** Lines 164-165

"Positive/negative" and "higher/lower" very confusing phrasing, with no means or statistics reported.

**Answer:** Lines 178-181

Within each group, the self-reported emotional valence for the positive and negative target emotions was significantly higher and lower, respectively, than for the Neutral State (*p* < 0.001; Neutral State valence mean ± SD, 4.0 ± 1.0 in the PD group and 3.8 ± 1.4 in the control group).

**Comment 9:** Figure 1

Numbers on bars would be easier to see if in white bold text, rather than black (or grey even).
Just to make abundantly clear, I would state in caption and/or y-axis label that concordance is the concordance between the intended target emotion and the experienced emotion.

**Answer:**

*We corrected the figure and caption.*

**Figure 1: Emotion self-reported scores in the Parkinson’s disease patients (N = 18) and healthy participants (N = 17).** Emotion concordance (bottom panel) is expressed as the percentage of individuals that did experience the intended target emotion. Emotion intensity (top panel) ranges between 0 and 6. Error bars represent the standard error of the mean. *χ^2^ = 8.15, *p =* 0.004; **ANOVA F = 5.33, *p =* 0.03; ***ANOVA F = 5.49, *p =* 0.03.

**Comment 10:** Line 192

Is this correlation analysis with an N of 18 or 28? Sample sizes would be helpful to report in much of your analysis as it is not always clear if the analysis involves the subgroups or the whole group.

**Answer: (**Line 194)

*We have now clarified in the text at the beginning of the paragraph that correlations between clinical and emotional data involved the whole PD group,* ***N = 28****. Clarification of N has been revised throughout the text.*

**Comment 11:** Line 244

Really interesting – the complexity of the emotion matters

**Answer:** Lines 322-328

In keeping with our findings though, a recent study investigating the ability of PD patients to recognize dynamic facial expressions reported a selective impairment in their capacity for processing cognitively complex emotions (*e.g.*, self-conscious emotions) conveyed by face cues but preserved basic facial emotion recognition [49]. Both the former and the present studies support that affective deficits in PD rather depend on the nature of the emotion and are more likely to manifest when more cognitive aspects of emotional processing are in demand.

**Comment 12:** Line 252

*have been, not has been

**Answer:** Line 267

*Corrected.*

**Comment 13:** Line 273

This sentence not grammatically complete, unless "related" is the verb, in which case confusing phrasing.

**Answer:** Lines 332-335

Urinary symptoms were the most common autonomic complaints of PD patients and their prevalence was associated with less intense and less pleasant feelings of Amusement, supporting the occurrence of non-motor features grouping in PD and possibly indicating a common
