# Supplementary material for: Beyond shallow feelings of complex affect: Non-motor correlates of subjective emotional experience in Parkinson’s disease
Source: PLoS One. 2023 Feb 24;18(2):e0281959. doi: 10.1371/journal.pone.0281959 (PMC9955984; doi:10.1371/journal.pone.0281959)
Supplement: Attachment — Submitted filename: Response to Reviewers.docx [file pone.0281959.s002.docx]

**Response to Reviewers**

**Reviewer #1**

**Beyond shallow feelings of complex affect: non-motor correlates of subjective emotional experience in Parkinson’s disease**

**This interesting study examines potential emotion-related abnormalities in Parkinson’s. The paper is extremely well-written and concise. It provides a clear rationale for the work. Whereas motor phenotypes have been fairly well-characterized in Parkinson’s, less is known about affective and autonomic dimensions of the disorder, though there is clearly evidence of costly emotional changes associated with the disease. Characterizing these emotional problems and potential subtypes may help inspire more targeted disease-modifying therapies.**

Thank you for the time devoted to evaluate the manuscript, we appreciate the helpful comments and all the recommendations given to improve the work. We have addressed in detail all the concerns. Please find below a line-by-line response to them.

**1) I always appreciate a concise introduction, but here I think this one could include a section on what is currently been done, and what the gaps are with respect to emotion in PDThere are studies on blunted reactivity (Bowers et al., 2006, Brain), reviews on emotion and PD (Sotjiu & Rusconi 2013 Front. Psychol; Peron et al., 2011 Movement disorders), emotion processing across different levels (Enrici et al., 2015 Plos one), reactivity to facial emotion (Ruzicka et al., 2019 Eur Neuropsychopharm), emotion detection and empathy (Martinez et al., 2018, Front Aging Neurosci). These are just a few.**

Following the reviewer’s suggestions, we have rewritten the paragraph in the Introduction that summarizes previous research on emotion in PD and included the recommended references, as follows:

(Lines 45-57) “Emotional disturbances are also part of the non-motor symptom complex of PD and concern all components of emotion [3]. Deficits in recognizing emotions from both the face and the voice, besides altered production of emotional facial expressions and speech, have been extensively documented in PD [4-8]. Likewise, impaired physiological responses to emotion have also been reported [9-12]. Considering different levels of affective processing, a previous study revealed deficits in the recognition and representation (attribution of a specific affective mental state) of emotion but no particular impairment in the regulation (management of one’s emotion) [13]. A later study also confirmed that PD patients have a decreased ability to detect emotion in others, which is associated with difficulty understanding another’s point of view (decreased empathy) and has important implications for caregiver burden [14]. Further research confirmed that some of these behavioral impairments are independent of cognitive function yet dependent on dopaminergic availability [15]. Lastly, emotion regulation impairment in the context of appropriate recognition and experience has also been informed [16].”

**2)Related to controlling for other factors:**

**a. There is virtually no information on the control group. Were they matched in terms of age, sex, and IQ? Did they do a BDI? Were they taking any psychotropic meds? What was the age of the PD group? A table would be helpful in this regard.**

IQ data was not collected in this study. We have now provided instead, information on the education level of participants, as it is related to IQ. The mean age of the whole PD group was 62.4 ± 9.0 years. A table with information on the age, sex, education level, BDI-II, and psychotropic medication in the experimental PD and control groups has now been added to the Results (Table 2, page 11). The experimental and control groups were homogenized in terms of age and sex and no significant differences in these demographic features were identified between them.

Table 2. Demographic and clinical characteristics of participants.

| **Feature** | **PD** | **Control** | **F/( χ^2^)** | ***p*** |
| --- | --- | --- | --- | --- |
| n | 18 | 17 | - | - |
| Sex (male:female) | 13:5 | 6:11 | (3.43) | 0.06 |
| Age (yrs) | 57.1 ± 7.2 | 55.0 ± 4.2 | 1.11 | 0.30 |
| Education level |  |  |  |  |
| Low (≤ 9 yrs) | 11 [2] | 0 | - | - |
| Medium (10 – 13 yrs) | 61 [11] | 24 [4] | - | - |
| High (≥ 14 yrs) | 28 [5] | 76 [13] | **(6.46)** | **0.01** |
| BDI-II (mean ± SD) | 7.4 ± 8.1 | 5.3 ± 6.8 | 0.60 | 0.45 |
| BDI-II (%) [n] | 22 [4] | 15 [2] | (0.00) | 0.99 |
| BDI-II, available n | 18 | 13 | - | - |
| Psychotropic medication | 5 | 1 | (1.61) | 0.20 |
| Anxiolytic | 2 | 1 | - | - |
| Antipsychotic | 1 | 0 | - | - |
| Antidepressant | 1 | 0 | - | - |
| Combination | 1 | 0 | - | - |

Values are expressed as mean ± standard deviation or percentage (number of subjects). ANOVA F or Pearson/Yates corrected χ^2^ with associated *p*-value are presented and appear in bold style for significant differences (*p* < 0.05). PD: Parkinson’s disease; n: number of cases; BDI-II: Beck Depression Inventory-II score.

The *effect of participant’s sex* was also further analyzed by a regression analysis and verified to have no significant influence on our findings. In the whole PD group (n = 28), sex had no a significant effect on Amusement intensity (t = 1.17, *p* = 0.25), Tenderness intensity (t = -0.08, *p* = 0.94) or Tenderness occurrence (t = 0.27, *p* = 0.79). Among participants form the experimental PD (n = 18) and control (n = 17) groups, sex did not significantly explain the differences in Amusement intensity (t = 1.57, *p* = 0.13), Tenderness intensity (t = 0.11, *p* = 0.92) or Tenderness occurrence (t = -0.09, *p* = 0.93)

It has been described that subjects with higher education have an increased risk of PD [1], although higher education might have protective effects in cognitive decline in PD [2]. We have verified however, by means of a regression analysis, that *education level* did not have a significant effect on the *emotion self-reported scores* of Amusement intensity (t = -0.02, *p* = 0.98), Tenderness intensity (t = 0.67, *p* = 0.51) and Tenderness occurrence (t = -0.33, *p* = 0.75) in the whole sample of PD patients (n = 28). In addition, we also confirmed that among participants from the experimental PD (n =18) and control (n = 17) groups, education level did not significantly explain the differences in Amusement intensity (t = -0.07, *p* = 0.94), Tenderness intensity (t = -0.31, *p* = 0.76) and Tenderness occurrence (t = 0.76, *p* = 0.45). Among the PD patients (n = 28), *education level* showed no significant effect on any of the *cognitive features* analyzed in this study, *e.g.*, MoCA score (t = 0.32, *p* = 0.75), except the Executive index score (t = 2.53, *p* = 0.02), which was considered in the analysis. Taken together, these results suggest that education level did not have a significant effect on the emotion and cognitive features of the study.

**References**

1. Frigerio R, Elbaz A, Sanft KR, Peterson BJ, Bower JH, et al. (2005) Education and occupations preceding Parkinson disease: a population-based case-control study. Neurology 65: 1575-1583.

2. Kierzynka A, Kaźmierski R, Kozubski W (2011) Educational level and cognitive impairment in patients with Parkinson disease. Neurol Neurochir Pol 45: 24-31.

**b. Participants were taking their usual dose of meds. Did they quantify this and look at associations with emotion? There are some systems that might be more sensitive to dopaminergic manipulations than others. It seems relevant.**

The possible impact of medication on the emotional response is a relevant issue. This was addressed by calculating the *levodopa equivalent daily dose (LEDD)* for each participant and assessing its correlation with the self-report scores of emotional experiences. Overall, we found no significant effect of dopaminergic medication (LEDD) on subjective emotional ratings. Only the occurrence, intensity and valence of Disgust were significantly correlated with the LEDD and levodopa equivalent dose at the time of the test (LED) (occurrence: LEDD R = -0.47, LED R = -0.41; intensity: LEED r = -0.40; valence: LEDD R = 0.43, LED R = 0.47).

A discussion on these results was provided for Amusement, Tenderness and Disgust:

(Discussion – Amusement, Tenderness. Lines 512-528)

“Concerning the affective motivation, another basic dimension of human affect, Tenderness, in contrast to Amusement, can be regarded as a high-approach motivational emotion [46,47]. Thus, a reduced occurrence of Tenderness may associate with a reduction of goal-directed behavior, a symptom of apathy. In contrast, a diminished intensity of both positive emotions may relate to a lowered ability to experience pleasure (anhedonia), a symptom of depression [48]. Still, in the present study, no significant effect of depression or psychotropic/dopaminergic medication was verified to explain the variance in participants’ ratings of emotional intensity and valence, similar to what has been previously reported [17,49]. These results suggest that the experience of Tenderness and Amusement may further depend on systems of neurotransmitters other than dopamine, compatible with models which propose that different contributions of neuromodulators, *i.e.*, dopamine, noradrenaline, serotonin, and acetylcholine, result in the more complex “higher-order” emotions [50,51]. In fact, equally high levels of noradrenaline and dopamine are found in the nucleus accumbens [52], suggesting that the noradrenergic system is implicated as well in mediating reward and motivation. In keeping with this, degeneration of both the locus coeruleus, the principal source of noradrenaline in the brain, and the nucleus accumbens has been related to motivational issues in PD that may be insensitive to dopamine medication [53,54].”

(Discussion – Disgust. Lines 673-677)

“Consistent with this, we found an inverse correlation between all aspects of Disgust subjective experience (occurrence, intensity and unpleasantness) and LEDD, possibly reflecting a link between reduced feelings of revulsion and greater olfactory dysfunction, which has been related to worse disease severity and, consequently, higher needs of antiparkinsonian medication [109].”

**c. On a related note, for some of the analyses, the authors controlled for the effect of disease duration. Did they take into account the severity of motor symptoms as well whilst on and off the medication? It sounds like they would have this data and it would be useful to examine the extent to which they covary with the emotional symptoms. If there is a dissociation, that would be of interest since it would argue for a subtype rather than an effect of disease severity.**

We have revised this aspect and modified the main analysis to consider also disease severity as suggested by the reviewer. Therefore, we performed a main multivariate analysis of covariance (MANCOVA), including both disease duration and severity as covariates to control their potential effect on the self-report emotional scores and clinical scales. Results of this analysis were updated in the Table 5 which shows the characteristics of the PD clusters. The PD patients in the present study were tested “ON” their normal dosage of dopaminergic medication.

We also verified through a correlation analysis that the severity of PD motor symptoms was not significantly associated with the self-reported scores of emotional experiences. The majority of patients included in the present study (62%, n = 18) had early PD (Hoehn & Yahr stage II).

**d. The issue concerning the potential effects of psychotropic medication is not yet adequately addressed. The medications taken by 10 of the patients are known to affect emotional reactivity. The authors note (line 149-150) that patients were 12 hours from their last intake; however, the half-life of some of these drugs is much longer (e.g., clonazepam 30-40 hours; olanzapine around 30 hours). The authors report the half-life of clonazepam as 8-12 hours (lines 390), but I think this is an underestimate (e.g., see DeVane et al., 1991, Psychopharm Bull; Crevoisier et al., 2003 Eur Neurol). It would be critical to present some data concerning whether those taking psychotropic meds showed the same pattern as those that did not.**

We have now removed the inaccurate statement about the duration of clonazepam’s effect.

Following the reviewer’s recommendations, we explored whether emotional scores differed between participants who were taking psychotropic medication and those who were not, by means of a regression analysis. In the whole sample of PD patients (n = 28), psychotropic medication did not affect Amusement intensity (t = -0.82, *p* = 0.42), Tenderness intensity (t = -0.98, *p* = 0.34) or Tenderness occurrence (t = -0.36, *p* = 0.72). In addition, among participants in the experimental PD (n = 18) and control (n = 17) groups, psychotropic medication did not explain the differences observed between both cohorts in Amusement intensity (t = -1.23, *p* = 0.23), Tenderness Intensity (t = -0.72, *p* = 0.47) or Tenderness Occurrence (t = -1.39, *p* = 0.17).

In the table below we present data showing that PD patients in the experimental group (PD n = 18) taking psychotropic medication (PD Psyc Med +, n = 5), namely clonazepam (CLZ), quetiapine (QTP) and amitriptyline (AMT), and some of those who were not taking these medications nor suffering from depression (PD Psyc Med -, n = 5) reported a comparable pattern of subjective scores for Amusement Intensity (Amus), Tenderness Intensity (Tend) and Tenderness Occurrence (Tend Occ). In fact, most of the individuals in the “PD Psyc Med -" sample reported worse subjective scores (*e.g.*, participants 1, 3 and 4) than most of those in the “PD Psyc Med +” sample. Median scores of emotional intensity were lower in the “PD Psyc Med -" than in the “PD Psyc Med +” sample (highlighted in red font).

Taken together, these results suggest that in the present study, psychotropic medication did not have a significant effect on the main findings.

| **PD Psyc Med +** | **Amus** | **Tend** | **Tend Occ** |  | **PD Psyc Med -** | **Amus** | **Tend** | **Tend Occ** |
| --- | --- | --- | --- | --- | --- | --- | --- | --- |
| **1 (CLZ)** | 3 | 3 | 0,5 |  | **1** | 2,5 | 2,5 | 0,5 |
| **2 (CLZ)** | 0,5 | 0 | 0 |  | **2** | 2 | 1 | 0 |
| **3 (QTP)** | 3,5 | 3,5 | 0,5 |  | **3** | 2,5 | 3,5 | 0,5 |
| **4 (AMT)** | 6 | 3 | 0 |  | **4** | 6 | 0 | 0 |
| **5 (CLZ + AMT)** | 1,5 | 3 | 1 |  | **5** | 1,5 | 6 | 0,5 |
| **Mean** | **2,9** | **2,5** | **0,4** |  | **Mean** | **2,9** | **2,6** | **0,3** |
| **Median** | **3** | **3** | **0,5** |  | **Median** | **2,5** | **2,5** | **0,5** |

This aspect has been summarized in the Results of the manuscript as follows:

(Lines 283-287) “Furthermore, depression (β1) and psychotropic drug medication (β2) in the PD patients did not show to have a significant influence on MoCA score (t_β1_ = 0.75, *p* = 0.46; t_β2_ = 1.13, *p* = 0.27), Tenderness occurrence (t_β1_ = 1.15, *p* = 0.26; t_β2_ = -0.45, *p* = 0.66) or intensity of Tenderness (t_β1_ = 0.24, *p* = 0.81; t_β2_ = -0.98, *p* = 0.34) and Amusement (t_β1_ = -0.44, *p* = 0.67; t_β2_ = -0.77, *p* = 0.45).”

**e. The results, conclusions and discussion should be revised in accordance with the above to ensure that they are in agreement with the data.**

Results, conclusions and discussion were revised and updated considering all these elements as detailed above.

**3)It is unfortunate that the intensity of the experienced emotion was rated on a 7-point scale, but the emotional valence was rated on a 9-point scale. To avoid confusion, making these the same would have been advantageous.**

We have acknowledged the fact that the emotional intensity and valence were rated using scales of different lengths as a limitation of the present study, as follows:

(Lines 755-756) “Moreover, it would have been advantageous to measure emotion intensity and valence using rating scales of similar size to facilitate their interpretation.”

**4)The introduction mentions autonomic arousal. In addition, physiological data were also collected during the experiment, but for some reason, they were not reported here. Given these data seem relevant (and provide additional information on emotional reactivity), it would seem they should also be reported here. The authors should provide a clear rationale for not presenting the data. I must admit, I have trouble thinking of a valid reason for omitting it.**

Some studies on emotional reactivity focus on specific aspects of emotion, *e.g.,* subjective experience, physiological arousal, facial/vocal expressions, or emotion recognition… In the present study, we aimed at assessing the subjective component of emotion and its association with other non-motor features relevant to PD. This allowed us to explore in depth and concentrate on specific deficits of PD patients related only to subjective attributes of emotion. We plan to evaluate other characteristics of PD emotional experience in separate studies. However, strong conclusions regarding aspects of physiological arousal would require considerably more analysis, which would significantly increase the extension and complexity of the present manuscript. Therefore, we only addressed in this work the subjective feeling aspect in order to be able to convey a clearer and concise message on its specific modification in PD. We have now updated the Introduction to emphasize that it is the *subjective component* *of emotion* what is the focus of the present work, as follows:

(Lines 58-67) “Despite the feeling aspect being an essential element of the concept of emotion, specific components of affective processing such as subjective emotional experience have received less attention in PD. Although previous research indicates that the attributes of subjective experience associated with emotion are preserved in PD [4,5,10,16-18], others have found that PD patients exhibit reduced ratings of subjective arousal in response to aversive or highly arousing pictures [9,19], as well as increased arousal ratings of emotional words reflecting “calm”, along with impaired valence ratings for words associated with positive and negative emotions [20]. Albeit the current evidence that the PD process does affect the subjective dimension of emotional experience, the limited existing studies have yielded inconsistent results. Thus, it is still unclear to what extent the ability to feel emotions is compromised in PD.”

(Lines 76-78) “Therefore, in the present study, we aimed to evaluate the subjective emotional experience of PD patients and its relationship with autonomic symptoms and other relevant non-motor manifestations.”

**5) I do not understand how the a set of features were identified for cluster analysis based on the description provided in lines 124-129. I was not sure whether there is a line missing or perhaps just more details are needed. A couple of lines here on what k means cluster analysis is and what it does would be helpful to readers as would a rationale for why it was applied with this data.**

We were interested in exploring the non-motor manifestations associated with the emotional deficits found in the present study. Therefore, were performed a correlation analysis in order to identify the most relevant associations between features. Based on these associations that were identified with the correlation analysis, we selected the features revealing emotional deficits in the PD patients (Amusement intensity and Tenderness occurrence) and those that resulted in having a significant correlation with them. We therefore included urinary symptom and MoCA scores to account for correlates of the emotional deficits suggested by reduced *Amusement intensity* and *Tenderness occurrence*, respectively.

We have provided further details on the clustering approach in the Methods section describing the statistical analysis and in the Results section, as follows:

(Methods – Statistical analysis. Lines 177-185)

“*Cluster analysis*. – Identifying the relevant correlations enabled appointing a set of features for clustering analysis (k-means) to further explore the non-motor correlates of emotional deficits in PD patients. Thus, features that suggested an impaired emotional experience and their most relevant correlates were used as input for the cluster analysis. This technique allows the exploration of the naturally occurring groups (clusters) within a dataset without any predefined labels or classes, as insights are derived from the data. To identify the optimal number of clusters, we applied the elbow method, based on the average degree of distortion measured in terms of Euclidean distances, combined with a recent approach proposed to unambiguously distinguish the elbow point by determining the cosine of interaction angle [36].”

(Results. Lines 326-331)

“*Cluster analysis.* – To further explore the clinical correlates of disturbances in subjective emotional experience in PD patients, we carried out a k-means cluster analysis, including as input the specific features suggesting emotional impairment, *i.e.*, Amusement intensity and Tenderness occurrence, and the non-motor aspects most strongly associated with them according to the correlation analyses, *i.*e., those from the autonomic (urinary SCOPA-AUT score) and cognitive (MoCA score) domains, resulting in four input variables.”

In the Introduction, we explicated that the clinical heterogeneity of PD suggest the existence of different subgroups. This is the rationale for further exploring the clustering of non-motor symptoms profiled according to the emotional deficits.

(Introduction fragment. Lines 79-86)

“PD is a complex disorder with wide variability in the clinical phenotype that suggests the existence of several subtypes of the disease [25]. Traditionally, two major motor clinical phenotypes have been emphasized, but little is known regarding the non-motor variability of PD. Therefore, exploring the link between the affective and autonomic dimensions may contribute to identifying sets of non-motor features that may be useful in delineating possible non-motor subtypes. Focusing on patient groups that share underlying pathological mechanisms is crucial for disease-modifying therapies to succeed and achieve precision medicine in PD.”

We assessed the validity of our resulting subgroups by exploring differences between them in other features that were not included as input in the cluster analysis (*e.g.*, autonomic complaints other than urinary, sleep, depression, motor laterality, other emotional scores…) and by verifying consistency between our clusters and previous descriptions of PD phenotypes, obtaining results that were in very strong agreement with the literature. In this regard, we were able to identify in our data the previously described phenotypes characterized by prominent non-motor involvement, *i.e.*, urinary symptoms (cluster 1), depression/thermoregulatory symptoms (cluster 2), RBD and amnestic cognitive syndrome (cluster 4), low non-motor involvement (cluster 5). Cluster 3 with prominent emotional deficits in subjective experience may be a novel finding of the present study.

**6) I would suggest some revisions to the organization of the results to make them more clear and the number of tests transparent. For example:**

**a. The presentation of results from the ANOVA conducted on the intensity ratings were hard to follow (lines 170-184). It would be simplest to describe what the ANOVA was exactly (e.g., was it a 2(Group: PD, control) X 2(Participant Sex) X 7(film emotion) first. Then describe each of the main effects Group, then sex, then film emotion. Then describe all interactions therein. Only those effects involving a significant interaction should then be unpacked with follow-up tests.**

The results on the ratings of subjective emotional experience were now reorganized as suggested by the reviewer and presented as follows (lines 257-292):

*Intensity*. – In general, the mean emotion intensity rated by the PD patients remained below the values reported by the healthy participants across the target emotions (group effect; multivariate one-way ANOVA F = 2.93, *p =* 0.03) and was significantly reduced for the experience of Tenderness and Amusement (Fig 1). In a complementary analysis, we verified that participants reported lower intensity for the subjective experience of Fear than for any other emotional experience (target emotion effect; 2 x 5 repeated measures ANOVA F = 10.05, *p* < 0.0001). PD patients reported the strongest feelings for Disgust, which scored among the least intense emotional experiences in the control participants. Yet, the target emotion and group interaction effect was not statistically significant (2 x 5 repeated measures ANOVA F = 2.24, *p =* 0.07).

*Valence*. – Emotional valence ratings were not significantly different between groups (group effect; multivariate one-way ANOVA F = 0.92, *p =* 0.50), although pleasant emotions (6.1 ± 1.2 in the PD group *vs.* 6.7 ± 0.9 in the control group; ANOVA F = 3.69, *p* = 0.06) exhibited a greater variation between groups than unpleasant emotions (1.5 ± 1.0 in the PD group *vs.* 1.4 ± 0.7 in the control group; ANOVA F = 0.12, *p* = 0.74). In a complementary analysis, we confirmed that the self-reported emotional valence for the positive and negative target emotions was significantly higher and lower than for the Neutral State (target emotion effect; 2 x 6 repeated measures ANOVA F = 145.99, *p* < 0.0001). The target emotion and group interaction effect was not statistically significant (2 x 6 repeated measures ANOVA F = 1.10, *p =* 0.36).

*Potential confounders.* – Multiple regression analysis was applied to consider the potential contribution of relevant confounders of subjective emotional experience. After controlling for the effect of the sex of participants (β1) from the experimental and control groups, we only confirmed a significant impact of PD (β2) on the occurrence of Tenderness (t_β1_ = -0.09, *p* = 0.93; t_β2_ = -2.43, *p* = 0.02) and the intensity of both Tenderness (t_β1_ = 0.11, *p* = 0.92; t_β2_ = -2.12, *p* = 0.04) and Amusement (t_β1_ = 1.57, *p* = 0.13; t_β2_ = -2.77, *p* = 0.009). In addition, we also confirmed that among these participants, the level of education did not significantly explain the differences in Tenderness occurrence (t = 0.76, p = 0.45), Tenderness intensity (t = -0.31, p = 0.76), and Amusement intensity (t = -0.07, p = 0.94). Furthermore, depression (β1) and psychotropic drug medication (β2) in the PD patients did not show to have a significant influence on MoCA score (t_β1_ = 0.75, *p* = 0.46; t_β2_ = 1.13, *p* = 0.27), Tenderness occurrence (t_β1_ = 1.15, *p* = 0.26; t_β2_ = -0.45, *p* = 0.66) or intensity of Tenderness (t_β1_ = 0.24, *p* = 0.81; t_β2_ = -0.98, *p* = 0.34) and Amusement (t_β1_ = -0.44, *p* = 0.67; t_β2_ = -0.77, *p* = 0.45). Finally, after controlling for the effects of participant’s sex and age where significant, the impact of PD on Amusement intensity (t = -2.30, *p* = 0.03) and Tenderness occurrence (t = -2.09, *p* = 0.04) was also confirmed in the whole sample of the study participants (n = 47), whereas the differences in Tenderness intensity were significantly explained only by the occurrence of this emotion (t = 2.14, *p* = 0.04).

**b. Lines 196-210: A number of correlations are presented. These should be presented in a table. It should also be made clear the number of correlational tests performed, the number of tests that were input for Benjamini Hochberg correction, and which correlations survive correction.**

Following this recommendation, we have now made clear this aspect in the Methods section describing the statistical analysis and the Results section, as follows:

(Methods – Statistical analysis. Lines 168-176)

“*Correlation analysis*. *–* The primary correlation analyses assessed the association between all self-reported scores of the target emotions that suggested an emotional impairment in PD and the nine scores describing the mood (depression), sleep, cognitive, urinary, gastrointestinal, cardiovascular, pupillomotor, thermoregulatory and sexual domains; comprising overall 54 tests. Pearson *r* or Spearman *R* correlation coefficients were determined and considered meaningful if *r* or *R* ≥ 0.45. The statistical significance was adjusted for multiple comparisons by controlling for the false discovery rate at *q*-value < 0.1, using the Benjamini-Hochberg (BH) correction. *Post hoc* analysis exploring the association between the variables from significant tests with the SCOPA-AUT scale, MoCA items, or other emotion scores was also considered.”

(Results. Lines 295-298)

“*Correlation analysis*. – We assessed the correlations between the clinical scales and the scores of occurrence, intensity, and valence reported for the two target emotions that suggested an impaired subjective feeling, *i.e.*, Amusement and Tenderness, in the whole group of PD patients (n = 28, Table 4).”

A table has now been added to summarize the correlation results (Table 4, page 16).

Table 4. Correlations between subjective emotional ratings and clinical scores in the Parkinson’s disease group.

| **Target emotion** | **Self-report score** | **SCOPA-AUT** | | | | **MoCA** | | | |
| --- | --- | --- | --- | --- | --- | --- | --- | --- | --- |
|  |  | Total | Urinary | GI | Sexual (men) | Total | Attention | Visuoexecutive |  |
| **Amusement** | Intensity | -0.26 | **-0.48** | 0.004 | -0.18 | -0.06 | 0.20 | -0.01 |  |
|  | Valence | **-0.51** | **-0.49** | -0.42 | -0.28 | -0.07 | 0.18 | -0.03 |  |
| **Tenderness** | Occurrence | **-0.45** | -0.32 | **-0.55** | -0.11 | **0.61** | **0.50** | **0.47** |  |
|  | Valence | -0.40 | -0.43 | -0.26 | **-0.70** | 0.18 | 0.20 | 0.31 |  |
| **Disgust** | Valence | 0.03 | 0.16 | -0.04 | **0.61** | -0.14 | -0.003 | -0.18 |  |

Strong correlations (correlation coefficient r ≥ 0.45) in the Parkinson’s disease group (n =28 patients, n = 19 males) that remained significant (*p*-value < 0.05) after adjusting for multiple comparisons by controlling for the false discovery rate at *q*-value < 0.1 appear in bold red style. Besides the MoCA items specified, Tenderness occurrence also correlated significantly with the Attention and Visuospatial indexes (Spearman R = 0.39 and R = 0.41, respectively). Higher and lower scores indicate more severe symptoms for the SCOPA-AUT and MoCA scales, respectively. GI: gastrointestinal; MoCA: Montreal Cognitive Assessment scale; SCOPA-AUT: Scales for Outcomes in Parkinson’s Disease-Autonomic

**c. I would suggest focusing on correlations between the experimental measures and the symptom measures. You could present correlations between clinical measures (e.g., MoCA and SCOPA-AUT and its subscales), but this is a separate issue and probably belongs in a different section. What is of greatest interest to the reader is the relationship between these symptoms and the experimental measures of emotional experience.**

We have revised the text of the manuscript and ensured that the focus has been placed on results describing the association between clinical scores and experimental measures of emotional experience as recommended by the reviewer.

**7)On line 259: there is suggestion that they found “no impairment in the subjective experience of basic emotions”. Could the authors clarify which metric determined this? If it is valence rating, it is important to consider effect sizes as I suspect that the issue might be one of power than that an effect is absence. One could do Bayesian approaches to assess the likelihood that the null is true if the authors want to make strong conclusions in this regard.**

We assessed the subjective experience of emotions by analyzing the occurrence, intensity and valence scores reported by participants in response to affective film clips. We agree with the reviewer in that it is important to consider the effect size as a potential element impacting the results. This aspect was acknowledged as a limitation of the present study, as follows:

(Lines 752-755) “A possibility is that a larger experimental group is necessary to increase the chances of detecting a significant effect of PD on hedonic tone, as valence ratings in response to overall pleasurable stimuli resulted in being slightly lower in the PD group (*p* < 0.06).”

**Reviewer #2**

**Dear Editors and Authors,**

**Thank you for the privilege of reviewing the manuscript titled “Beyond shallow feelings of complex affect: non-motor correlates of subjective emotional experience in Parkinson's disease”. In it, the authors seek to evaluate the affective changes in Parkinson’s disease (PD) by examining subjectively reported emotional responses to movie clips. They also seek to determine the relationship between non-motor/autonomic symptoms of PD, and these emotional responses.**

**I generally thought this was an interesting, and well written paper, and would support its publication so long as some of the issues below can be addressed.**

**In particular, the issue regarding the possible impact of the long-lasting benzodiazepines used by many of the PD patients in this study needs to be further discussed or analyzed, given that these medications can possibly affect emotional response, and that a study which found different results that this paper (citation 9) did not have any patients on these medications. As well, I will ask the Editor to ensure that either another reviewer, or external statistical expert, is able to determine the validity of the statistical analysis used, as they are beyond my expertise.**

**Strengths**

**1.I found the paper generally very well written and easy to follow.**

**2.I think this is an interesting and helpful topic to further understand, and a good application of the methodologies used**

**3.The authors do a good job discussing their results and interpreting it with respect to prior literature.**

**4.Studying a non-native English speaking population helps add needed diversity to the literature.**

**Major Issues**

**1.My computational expertise are more in machine learning methodology than in statistics. While I generally thought the statistical analysis was done correctly, I will ask the editor to ensure either an outside expert, or another reviewer with such expertise, is able to confirm the validity of the statistical methods used, especially given the complexity and relatively small sample size.**

We thank the reviewer for the thorough evaluation of the manuscript and important recommendations, which lead to improving the work and notably refining our findings.

**2.K-means requires that users specify the number of clusters they wish to apply the algorithm to. From what I can tell, the authors choose 4 clusters given prior work that used this number (citation #20). Looking at this reference, it seems there was some similarity in the data. I’d ask that the authors please further validate why using this number is okay – is that prior work’s dataset/population so similar that this number can be used again? Have other work also used this number? Otherwise, it might be preferable to do some analysis yourselves that this is an optimal number of clusters (as was done in citation 20).**

Following the reviewer’s recommendation, for identifying the optimal number of clusters, we applied the elbow method based on the average degree of distortion measured in terms of Euclidean distances, in combination with a recent approach proposed to unambiguously distinguish the elbow point by determining the cosine of interaction angle:

- Shi, C., Wei, B., Wei, S. et al. A quantitative discriminant method of elbow point for the optimal number of clusters in clustering algorithm. J Wireless Com Network 2021, 31 (2021). https://doi.org/10.1186/s13638-021-01910-w

According to this analysis, we found that five clusters were a better solution for our data than four clusters.

Optimal *elbow point*, after which the distortion starts decreasing in a linear fashion with further increases of k.

k_opt_ = k of minimum (α_3_, α_4_, α_5_)

k_opt_ = 5

α_3_ = 2.90

α_4_ = 2.97

α_5_ = 2.82

Addressing this important concern, allowed us to obtain much more delineated clusters. Thus, we were able to identify five PD profiles highly compatible with those previously described in the literature, which are characterized by prominent and specific non-motor manifestations, namely:

- cluster 1: urinary and non-amnestic “fronto-striatal” syndrome; impaired Amusement and Tenderness,
- cluster 2: autonomic dysfunction (greater involvement of thermoregulatory and pupillomotor issues) and depression; Amusement selectively impaired,
- cluster 3: impaired visuospatial abilities and subjective feeling (several emotional alterations),
- cluster 4: probable RBD and amnestic cognitive deficits; Tenderness selectively impaired, and
- cluster 5: lowest non-motor involvement and lowest dose of dopaminergic treatment; no significant emotional deficits

Figure 2 has now been added to summarize the non-motor characteristics of the PD profiles revealed in the present study. Cluster 3, displaying the greatest modifications in subjective emotional experience, some of which has been previously reported, may be a novel finding of the present study. These patients (n = 6) were reassigned, four non-depressed patients from former cluster 2 (current cluster 2) and two right-sided motor onset patients from former cluster 3 (current cluster 4), while the former clusters 1 and 4 remained intact (current cluster 1 and 5, respectively). Among the emotional deficits revealed in this cluster, higher intensity of Fear and higher valence of Disgust have both been previously reported in PD, and associated with olfactory impairment or dopamine overdose. Please note the updates made to the Discussion in order to elaborate on these new findings (lines 572-740).

**3.A larger concern I have regarding the validity of the results lies in the impact of the subject’s pharmacology. 10 patients are taking psychotropic medication. However, the authors mention that “Excluding the four patients under antidepressant and antipsychotic treatment from the analysis did not alter these results confirming no significant effect of the psychotropic drugs on the present findings” and in the limitations note “however, since [clonazepam’s] effects last about 8 to 12 hours, no important influences on the study measures were expected”. My understanding and experience as a psychiatrist is that while the acute anxiolytic/sedative effects might be though to last 8 to 12 hours, they do not stop having any psychotropic effect after this period. As well, did subjects stop taking their medications more than 12 hours before the testing? Clonazepam has a half-life 19-60 hours, so at steady state is still going to be at high serum levels even 12 hours last dose. It is sometimes used in anxiety and epilepsy disorders being prescribed once daily. The authors cite that it is not known to have an effect on REM sleep parameters. However, my understanding is it can affect cognition, especially in the elderly, as well as emotions. I believe the authors must better establish that clonazepam/alprazolam use is not contributing to the differences found in the study. This is especially important given the relatively large number of participants on them, and given that the differences found were in more complex emotions, which could be affected by the cognitive-effects of these long-lasting benzodiazepines in this elderly population. Analysis of PD subjects without those on any of the psychotropic medications (or not on a benzo) would be helpful, or further citation of prior work showing that these medications would not be expected to affect the complex emotional responses like tenderness. I note that in citation 9, where they found that the emotion response in PD was unimpaired, none of the subjects seem to have been on benzodiazepines, though some are on antidepressants; citation 11 does not seem to discuss this. Citations 10 and 20 seemed to exclude patients on psychotropic medication or with psychiatric history I think, though this may not be naturalistic.**

We have now removed the inaccurate statement about the duration of clonazepam’s effect.

In order to address this important concern, we carried out a regression analysis and verified that psychotropic medication (psycMed) did not have a significant effect on cognition (MoCA score) in the PD patients (n = 28, t = 1.20, *p* = 0.24). Moreover, the highest prevalence of psycMed (50%, n = 2) was found in cluster 2, also exhibiting the best cognitive score (MoCa = 27.5 ± 1.3), whereas the lowest prevalence of psycMed (20%, n = 1) was found in cluster 4, showing the worse cognitive status (MoCA = 21.6 ± 1.1).

For considering the effect of psychotropic medication on emotion, please see our detailed response to a similar concern of Reviewer #1 – in “Comment **2) d.**”. In the presented table, data of PD patients who are not taking psychotropic drugs is depicted and compared to those on medication, finding similar results.

**4.Somewhat related to the above, I’d ask that the authors further address the possible impact of depression on the results, especially if the patients had a long, lifetime history of depression which preceded the PD. Could depression not attributable to PD be explaining some of the results found by the authors? Prior literature, or analysis of those without depression, could be helpful to ensure that PD is the cause of the findings. I do recognize that PD can cause depression, of course, but I think some more consideration would be helpful to help tease this apart, though I acknowledge the authors did do this to some extent by excluding those on antidepressants and finding the same results.**

After carefully revising this concern, the following results suggest no significant effect of depression on the main findings of the study:

- We carried out a regression analysis and verified that depression did not have a significant effect on scores of Amusement intensity (t = -0.50, *p* = 0.62), Tenderness intensity (t = 0.16, *p* = 0.87), or Tenderness occurrence (t = 1.14, *p* = 0.27) in the sample of PD patients (n = 28). This results suggest that depression had no a significant effect on these emotion scores among the PD patients.
- We also confirmed through regression analysis that depression did not explain the differences between the experimental (PD n = 18) and control (n = 13) groups in Amusement intensity (t = -0.99, *p* = 0.33), Tenderness intensity (p = 0.22, *p* = 0.83), or Tenderness occurrence (t = 0.26, *p* = 0.80).
- In cluster 2 with a high prevalence of depression (75%), mean intensity of Tenderness (4.5 ± 1.5) was comparable to that of controls (4.4 ± 1.2), whereas Tenderness occurrence (0.9 ± 0.3) was higher on average. This result corroborates that depression is not associated with an impaired experience of Tenderness in PD.
- Cluster 3 included no patient with depression; yet in this cluster, patients showed the greatest overall impairment in subjective emotional experience, especially deficits in Amusement feeling. This result suggests that PD patients with no depression may exhibit a significant reduction in Amusement intensity, as well as Tenderness occurrence.
- The figure contrasts the characteristics of subjective emotional experience in a cluster with depression (cluster 2, n = 4) and another one without it (cluster 3, n = 6). This suggest that impaired feeling of Tenderness (occurrence and intensity) and Amusement (intensity) is a feature of PD irrespective of the depressive status. In fact, the occurrence and intensity of Tenderness and Amusement were higher in the cluster including depressed patients.
- Of note, the non-motor PD phenotype with dominant autonomic impairment (thermoregulatory dysfunction) and high levels of depression has been previously described:
- van Wamelen DJ, Leta V, Podlewska AM, Wan Y-M, Krbot K, et al. (2019) Exploring hyperhidrosis and related thermoregulatory symptoms as a possible clinical identifier for the dysautonomic subtype of Parkinson’s disease. Journal of Neurology 266: 1736-1742.

Furthermore, the association between autonomic and depression symptoms in PD has been also emphasized by others and suggested to reflect overlapping physiology:

- Sklerov M, Browner N, Dayan E, Rubinow D, Frohlich F (2022) Autonomic and Depression Symptoms in Parkinson's Disease: Clinical Evidence for Overlapping Physiology. J Parkinsons Dis 12: 1059-1067.
- Kim JY, Song IU, Koh SB, Ahn TB, Kim SJ, et al. (2017) Validation of the Korean Version of the Scale for Outcomes in Parkinson's Disease-Autonomic. J Mov Disord 10: 29-34.
- Zhang F, Wang F, Li C-H, Wang J-W, Han C-L, et al. (2022) Subthalamic nucleus-deep brain stimulation improves autonomic dysfunctions in Parkinson’s disease. BMC Neurology 22: 124.

**Minor Issues**

**1.“The sex of participants had not a significant effect on the differences observed in the mean emotion intensity reported for these target emotions” I would suggest “The sex of participants did not have a …” as the current wording reads a bit odd to me.**

This has been revised and corrected.

**2.Table 2 – for the drugs, there are percentage signs in the reported numbers, but not for other percentages reported.**

This has been revised and corrected.
